# Supplementary material for: REEP5 depletion causes sarco-endoplasmic reticulum vacuolization and cardiac functional defects
Source: Nat Commun. 2020 Feb 19;11:965. doi: 10.1038/s41467-019-14143-9 (PMC7031342; doi:10.1038/s41467-019-14143-9)
Supplement: Supplementary file 1 — Supplementary Information [file 41467_2019_14143_MOESM1_ESM.pdf]

REEP5 depletion causes sarco-endoplasmic reticulum vacuolization and cardiac functional defects

Lee and Hadipour-Lakmehsari *et al.*

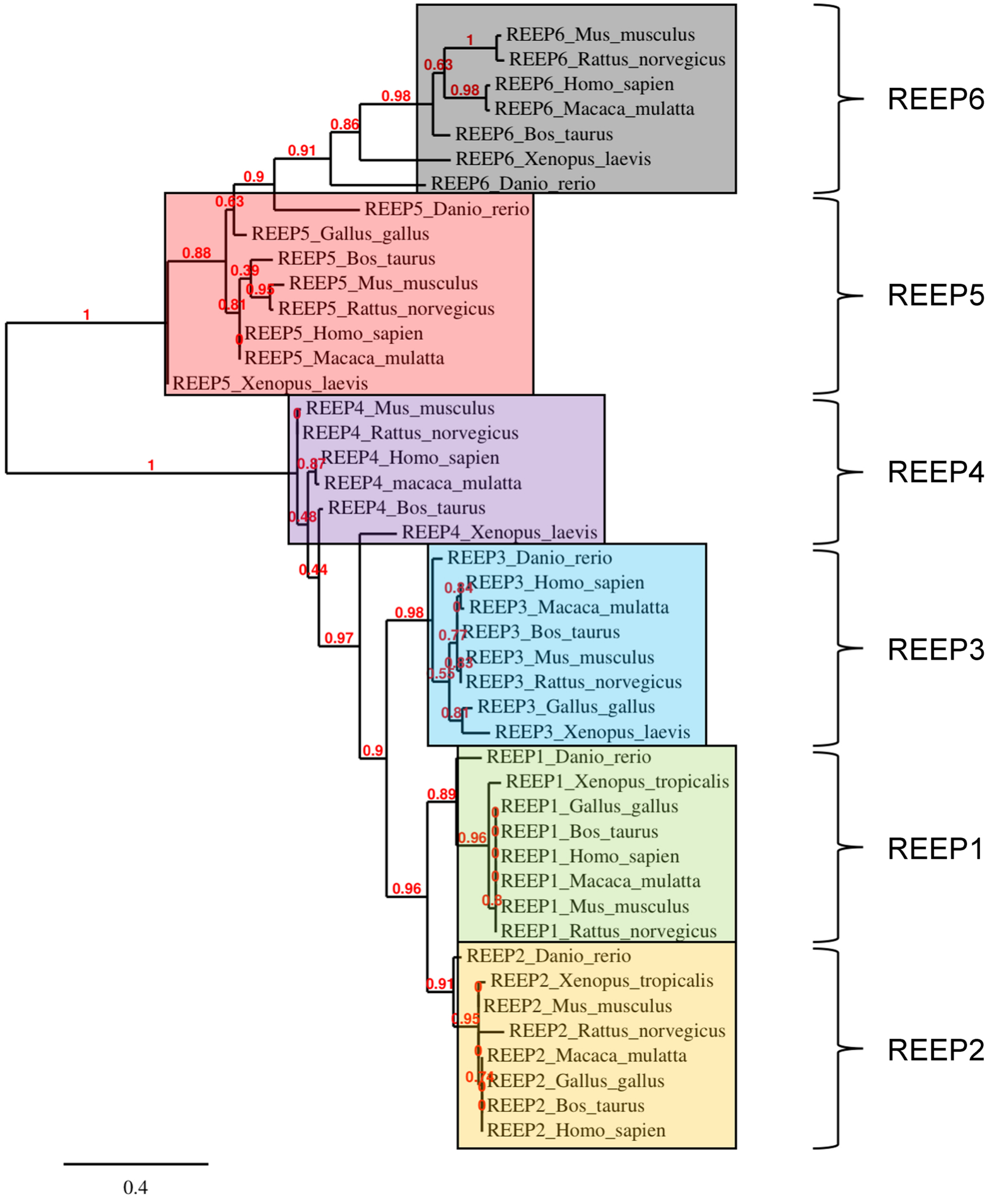

Supplementary Figure 1. Phylogenetic analysis of the REEP family of proteins using Phylogeny.fr (www. phylogeny.fr) demonstrates clustering of mammalian taxa with conservation throughout multiple species. Analysis identifies two phylogenetically distinct groups: REEP1-4 and REEP5-6.

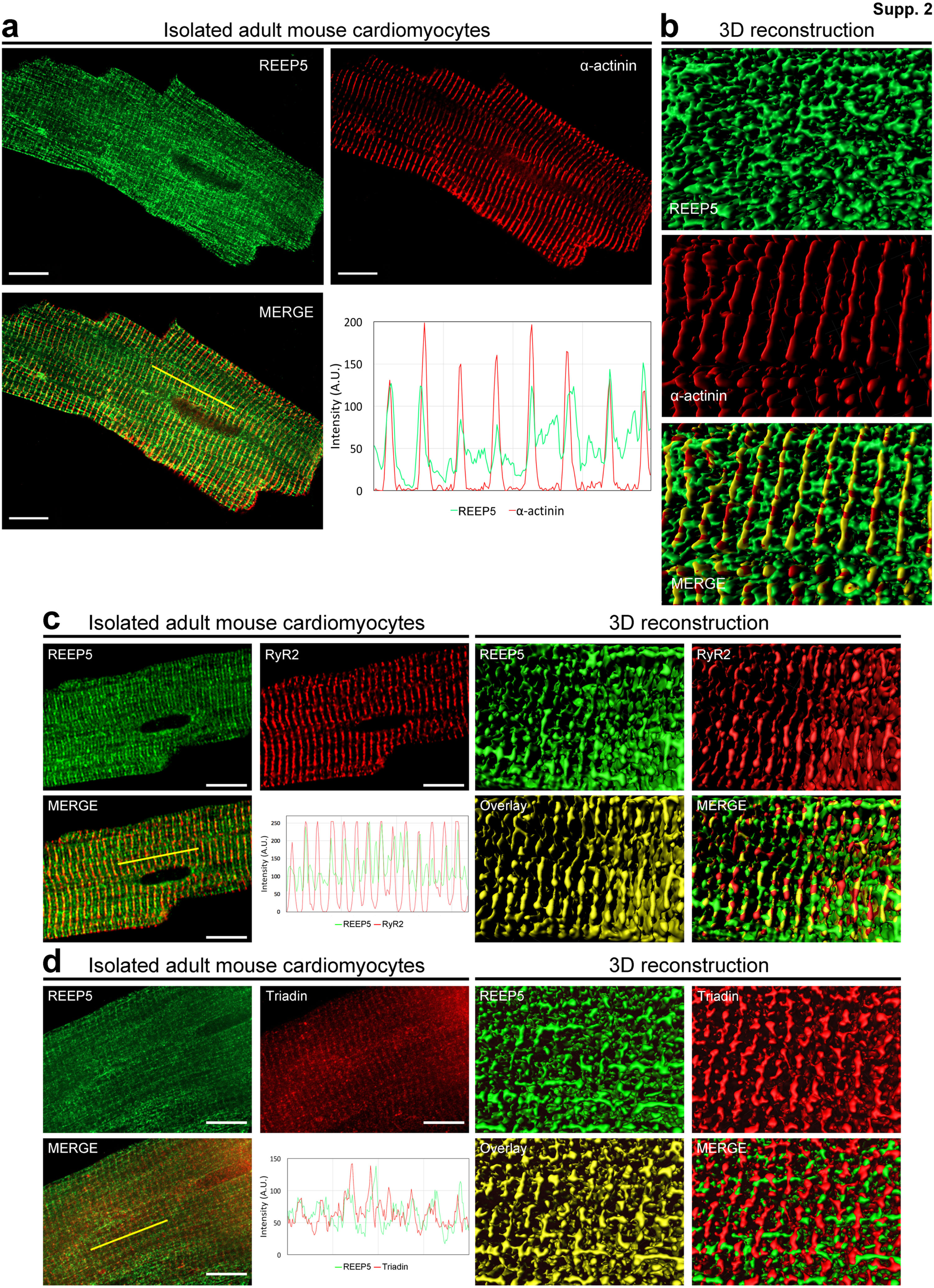
a
Isolated adult mouse cardiomyocytes
REEP5
α-actinin
MERGE
200
150
100
50
0
Intensity (A.U.)
REEP5
α-actinin
b
3D reconstruction
REEP5
α-actinin
MERGE
c
Isolated adult mouse cardiomyocytes
3D reconstruction
REEP5
RyR2
MERGE
Overlay
250
200
150
100
50
0
Intensity (A.U.)
REEP5 RyR2
d
Isolated adult mouse cardiomyocytes
3D reconstruction
REEP5
Triadin
MERGE
Overlay
150
100
50
0
Intensity (A.U.)
REEP5 Triadin

Supplementary Figure 2. REEP5 immunostaining demonstrates SR staining pattern and co-localization with known j-SR proteins, α-actinin, RyR2, and triadin in cardiac myocytes. (**a**) Immunofluorescence of acutely isolated adult mouse cardiac myocytes with REEP5 (green) co-stained with cardiac sarcomeric α-actinin (red). Line scan analysis of yellow bar shows co-localization between REEP5 and α-actinin at the j-SR. Scale, 10μM. (**b**) Three-dimensional reconstructive analysis (Imaris) demonstrates 56.78%±1.29% area of co-localization (overlay) between REEP5 and α-actinin with a Pearson coefficient of 0.61±0.04. Data presented as mean±SEM. (**c**) Immunofluorescence and three-dimensional reconstructive analyses of acutely isolated adult mouse cardiac myocytes with REEP5 (green) co-stained with RyR2 (red). Scale, 10μM. (**d**) Immunofluorescence and three-dimensional reconstructive analyses of acutely isolated adult mouse cardiac myocytes with REEP5 (green) co-stained with triadin (red). Scale, 10μM. All images shown are representative of approximately 30-40 total images captured per condition, n=3 independent biological replicates.

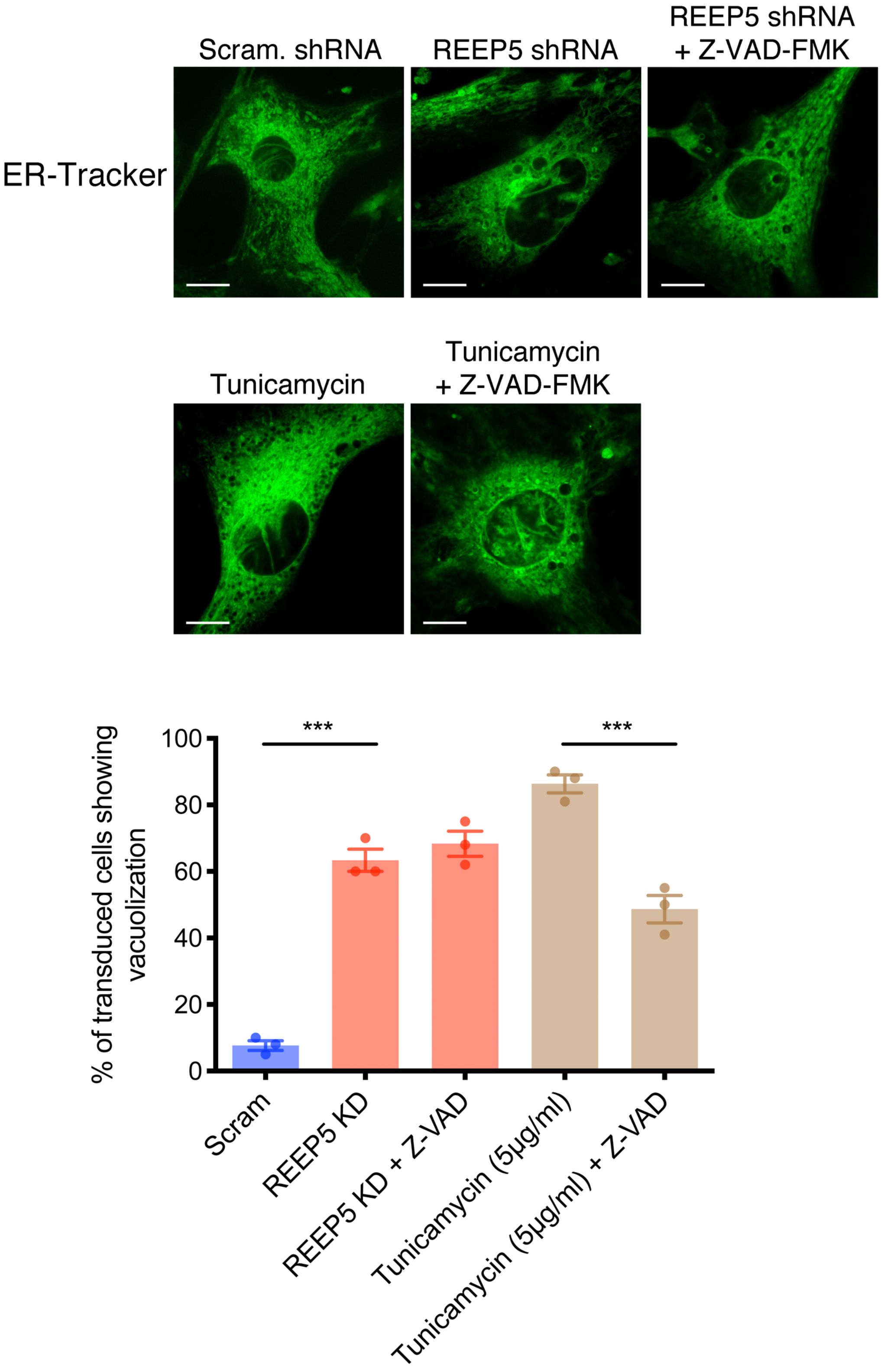

Supplementary Figure 3. REEP5 depletion-induced SR/ER vacuolization is independent of cellular apoptosis in cardiac myocytes. Confocal imaging of REEP5 shRNA infected CMNCs stained with ER-Tracker shows consistent SR/ER luminal vacuoles in the presence of caspases inhibitor z-vad-fmk. Treatment of z-vad-fmk significantly reduced ER stress-mediated SR/ER vacuolization, indicating distinct cellular mechanisms involved in REEP5 depletion-mediated SR/ER vacuolization versus prolonged ER stress-mediated cytoplasmic vacuolization. All images shown are representative of 30 total images captured per condition, n=3 independent biological replicates. Scale, 10µM. Quantitative analysis was done under 40x objective lens and approximately 30-40 cells were scored and data averaged for each experimental condition. Asterisks indicate a statistically significant *p* value in a tukey's multiple comparison analysis where ***$p<0.001$; data are presented as mean±SEM.

a REEP5-REEP5-EYFP

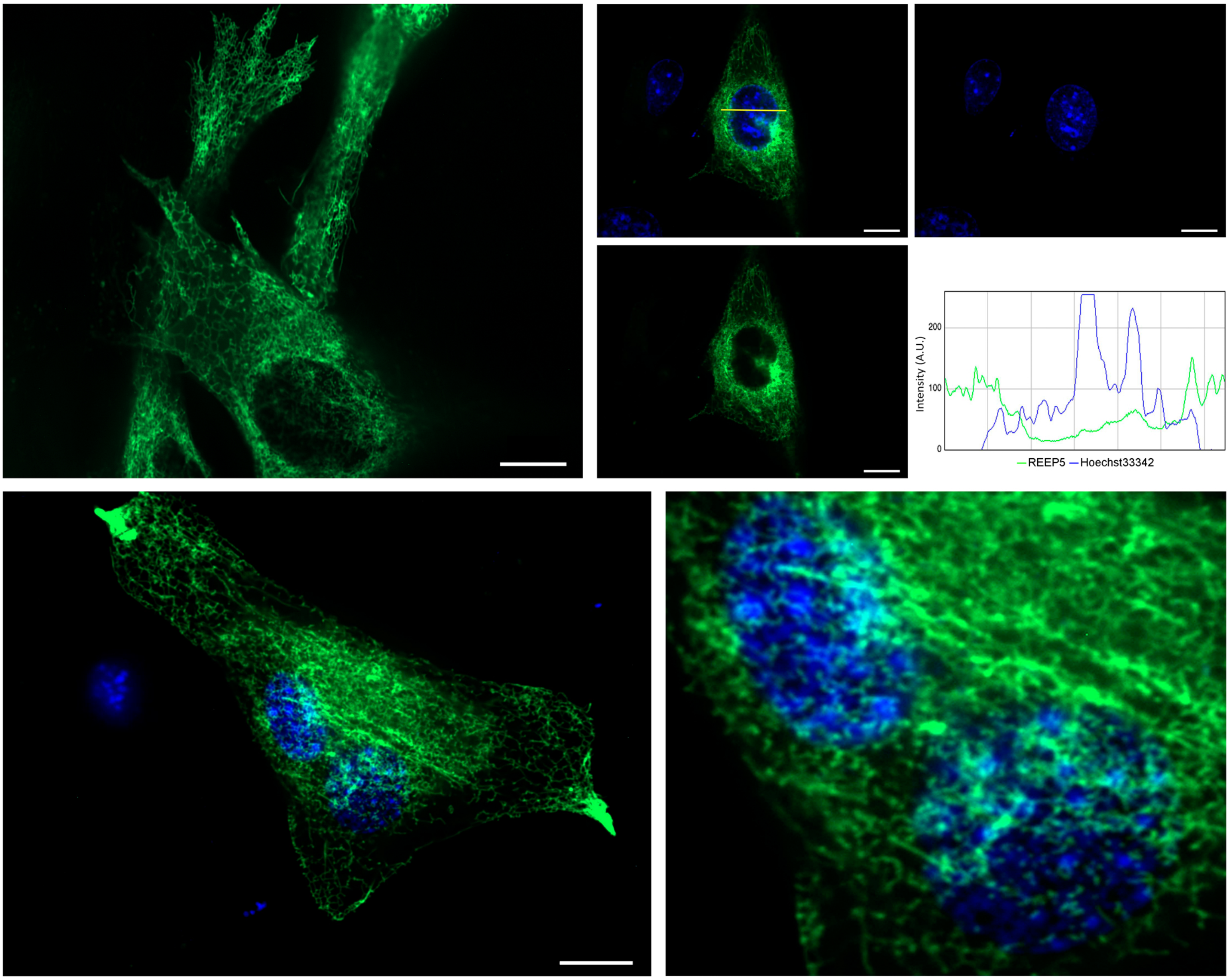

b Δ114-189 REEP5-EYFP / mCherry-α-Tubulin

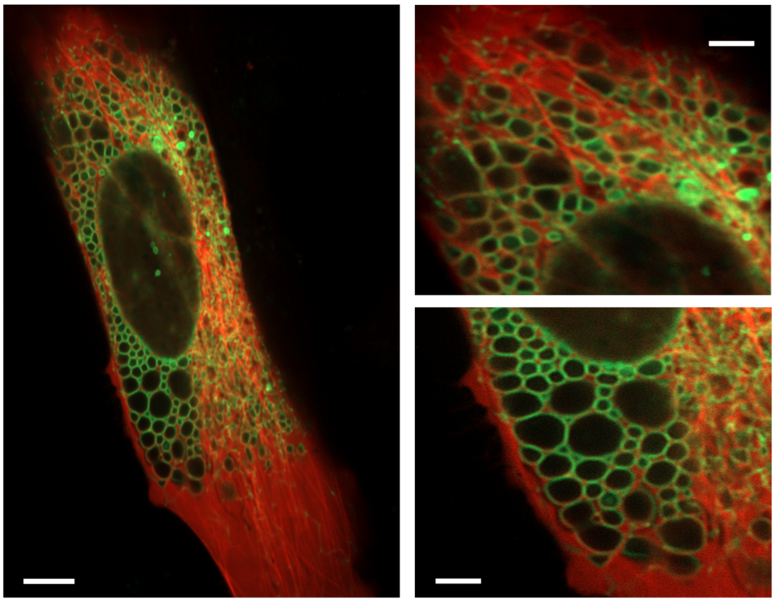

Supplementary Figure 4. Overexpression of dimerized REEP5 robustly marks the ER network in mouse myoblasts. (**a**) Confocal imaging shows dimerized REEP5 extensively marks the ER network in C_2C_{12} myoblasts transfected with REEP5-REEP5-EYFP. Scale, 20μM (left), 10μM (right). (**b**) Confocal imaging of C_2C_{12} myoblasts co-transfected with Δ114-189 REEP5-EYFP and mCherry-α-Tubulin. Scale, 20μM (left), 10μM (right). All images shown are representative of 30-40 total images captured per condition, n=3 independent biological replicates.

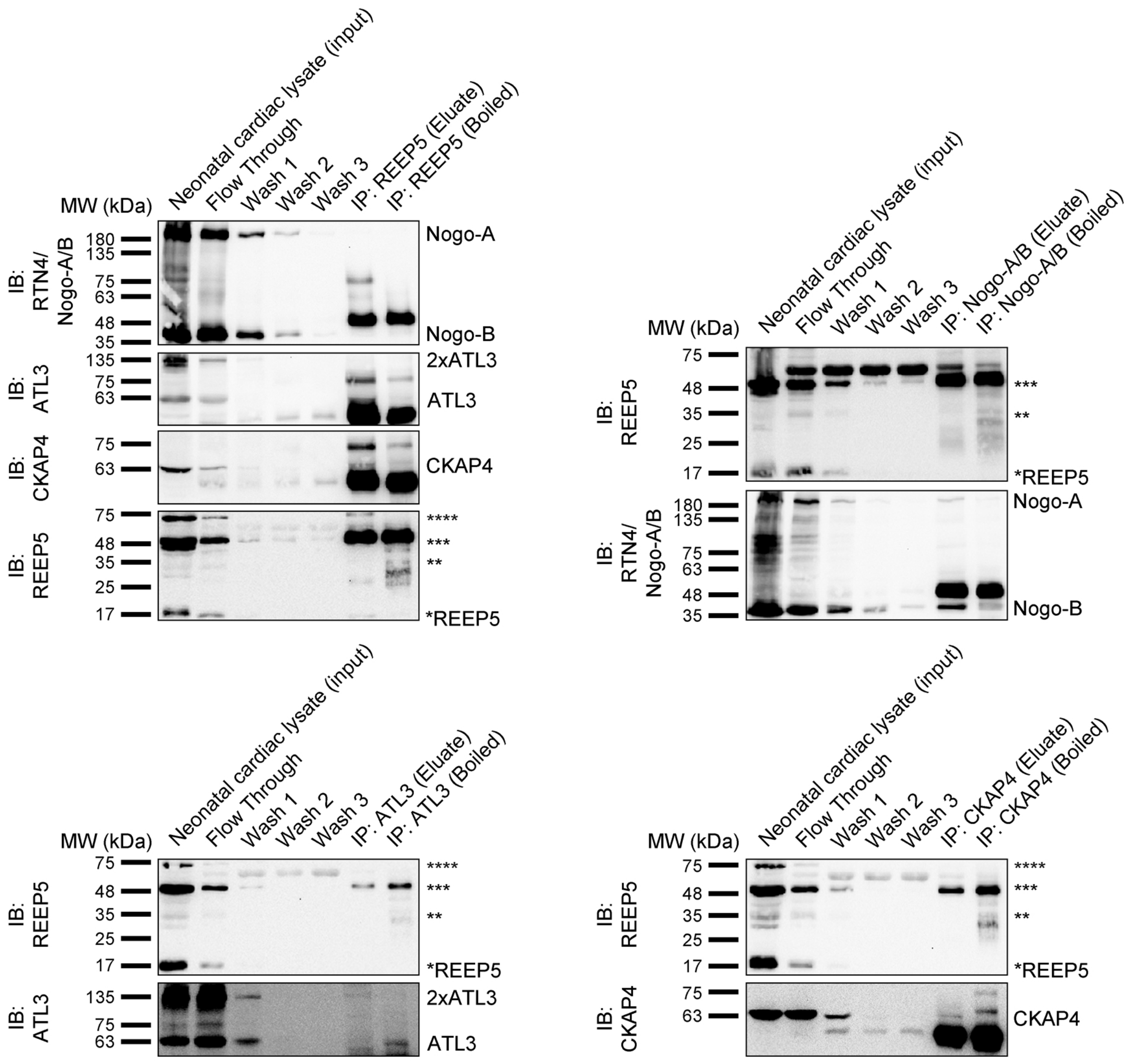

Supplementary Figure 5. Co-immunoprecipitation assays demonstrate dynamic REEP5 interactions with RTN4/Nogo A/B, ATL3, and CKAP4 in neonatal ventricular cardiac lysate. Co-immunoprecipitation and reverse-order co-immunoprecipitation with anti-REEP5, anti-Nogo A/B, anti-ATL3, and anti-CKAP4 antibodies in neonatal ventricular cardiac lysate (input), n=3 independent biological replicates. Asterisks to the right indicate the number of predicted REEP5 monomer, dimer, trimer, and oligomer detected based on anticipated molecular weight. Source data containing original uncropped immunoblots are provided as a Source Data file.

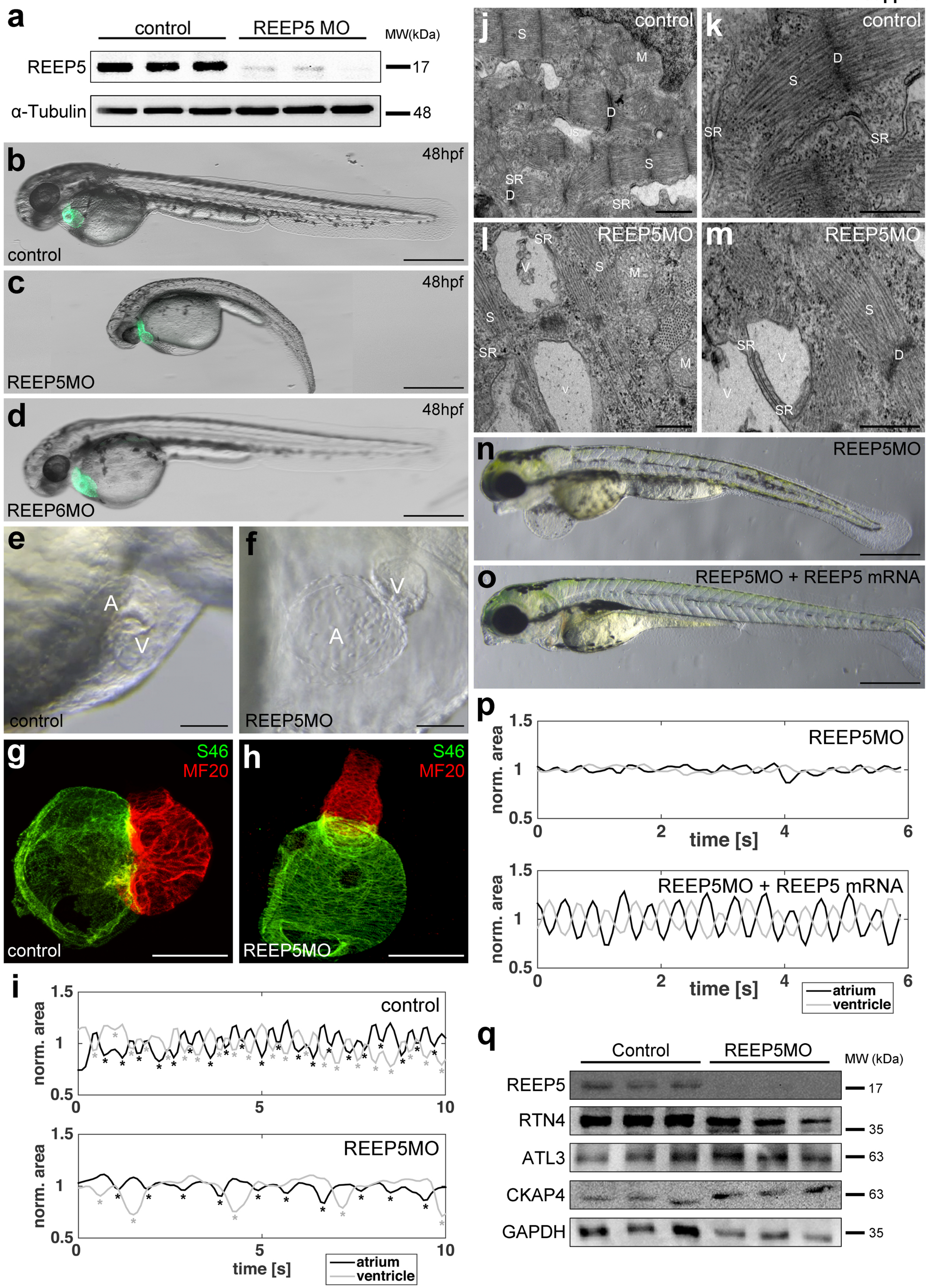
a
control
REEP5 MO
MW(kDa)
REEP5
17
α-Tubulin
48
b
48hpf
control
c
48hpf
REEP5MO
d
48hpf
REEP6MO
e
A
V
control
f
V
A
REEP5MO
g
S46
MF20
control
h
S46
MF20
REEP5MO
i
control
norm. area
time [s]
REEP5MO
atrium
ventricle
j
control
k
control
l
REEP5MO
m
REEP5MO
n
REEP5MO
o
REEP5MO + REEP5 mRNA
p
REEP5MO
norm. area
time [s]
REEP5MO + REEP5 mRNA
atrium
ventricle
q
Control
REEP5MO
MW (kDa)
REEP5
RTN4
ATL3
CKAP4
GAPDH
17
35
63
63
35

Supplementary Figure 6. Morpholino-mediated REEP5 depletion in zebrafish embryos causes severe developmental abnormalities. (**a**) Immunoblot analysis of REEP5 expression at 48hpf in wild-type and REEP5 MO injected embryos. (**b-d**) *Tg(myl7:EGFP)* transgenic embryos injected with 1ng REEP5 and REEP6 MOs and imaged at 48hpf reveal overt cardiac defects in REEP5 MO embryos and no observable cardiac phenotype in REEP6 MO embryos. Scale, 500µm. Images shown are representative of 43 control, 68 REEP5 MO, and 31 REEP6 MO total injected embryos, n=3 independent biological crosses. (**e,f**) Optical imaging of REEP5 MO embryos reveals cardiac looping defects and swelling of the atrium, ventricle, and the pericardial sac compared to fully-looped control embryo hearts. A, atrium; V, ventricle. Images shown are representative of 63 control and 84 REEP5 MO total injected embryos, n=3 independent biological crosses. Scale, 10µm. (**g,h**) Immunofluorescence analysis of atrial (green) and ventricular (red) chambers at 72hpf shows arrested heart looping process in REEP5 MO hearts. Scale, 10µm. (**i**) Movie imaging analysis showing irregular cardiac beating rhythms with reduced beating frequency and dyssynchronous atrioventricular contractions in REEP5 deficient embryos. Area profiles were smoothened with a Gaussian curve with σ=0.2s. (**j**) TEM of control zebrafish ventricular tissue showing intact ventricular myocardium. Scale, 1µm. (**k**) Higher magnification of control zebrafish ventricular myocardium showing intact sarcomeres and SR. Scale, 500nm. (**l**) TEM of REEP5 MO zebrafish ventricular tissue showing SR vacuolization and disorganized muscle fibers. Scale, 1µm. (**m**) Higher magnigication of REEP5 MO ventricular myocardium showing vacuolated SR. Scale, 500nm. S, sarcomere; M, mitochondria; SR, sarcoplasmic reticulum; D, desmosomes; JS, junctional space; V, vacuole. (**n,o**) REEP5 mRNA rescue studies co-injecting REEP5 mRNA and REEP5 MO effectively rescued the aberrant heart morphology observed in the REEP5 morphant embryos. Scale, 500µm. (**p**) Movie imaging analysis showing corrected cardiac beating rhythms with normal atrioventricular contractions in the rescued embryos. Area profiles were smoothened with a Gaussian curve with σ=0.2s. (**q**) Immunoblot analysis of cardiac-enriched ER structure proteins (RTN4, ATL3, and CKAP4) in REEP5 MO mutant hearts, n=3 independent replicates from three independent biological crosses.

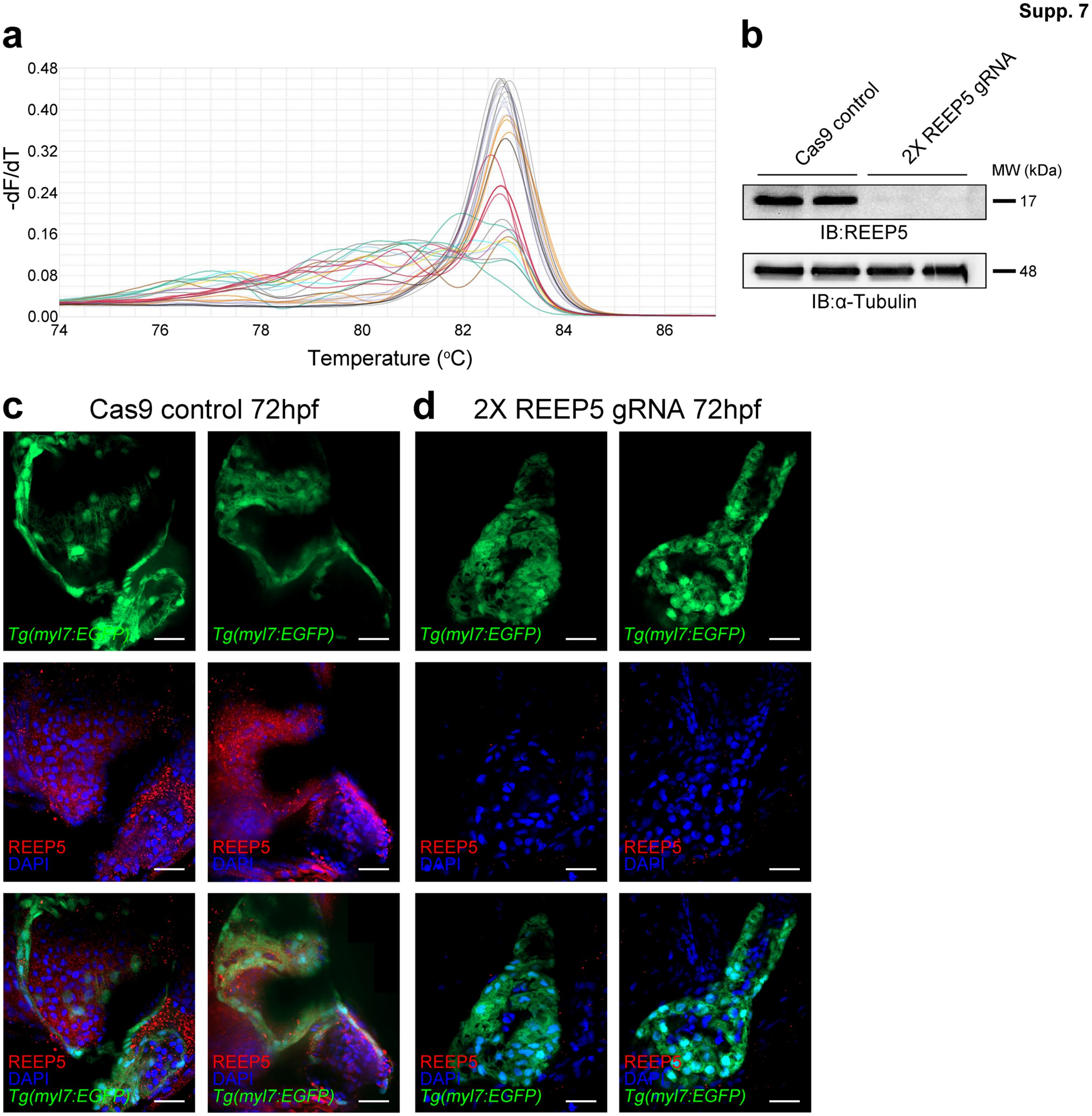

Supplementary Figure 7. CRISPR/Cas9-mediated REEP5 loss-of-function crispants demonstrate cardiac developmental and functional defects in zebrafish embryos. (**a**) HRM analysis of REEP5 gRNA injected embryos shows efficient cutting of the *reep5* gene in zebrafish. (**b**) Immunoblot analysis of REEP5 and α-Tubulin levels in control and 2X REEP5 gRNA injected embryos. Source data containing original uncropped immunoblots are provided as a Source Data file. (**c,d**) Immunofluorescence analysis of REEP5 expression in control and 2X REEP5 gRNA injected *Tg(myl7:EGFP)* transgenic embryo hearts shows depletion of REEP5 expression and linearized hearts associated with REEP5 gRNA injection. Scale, 50μm. All images shown are representative images of approximately 30 total images captured per condition, n=3 independent biological replicates.

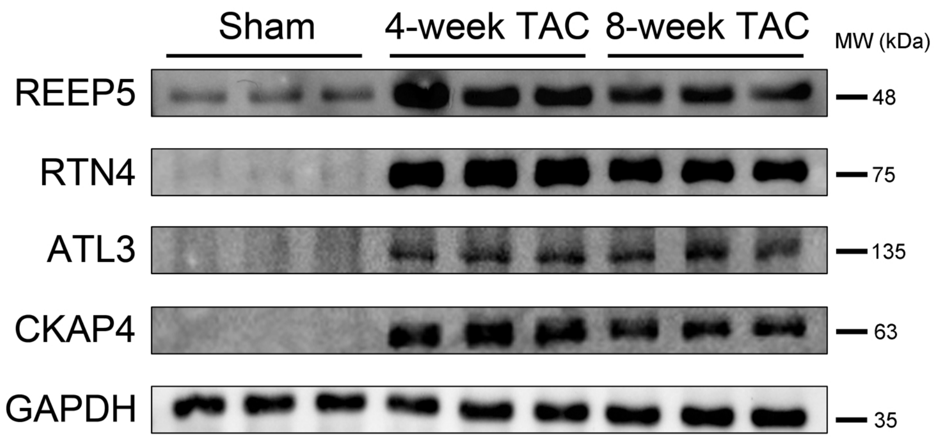

Supplementary Figure 8. Increased protein expression levels of REEP5, RTN4, ATL3, and CKAP4 in pressure overload-induced mouse failing hearts. Immunoblots of REEP5, RTN4, ATL3, and CKAP4 in mouse failing hearts at 4-week and 8-week post-transverse aortic constriction, n=3 independent biological replicates. Source data containing original uncropped immunoblots are provided as a Source Data file.
