# Supplementary material for: REEP5 depletion causes sarco-endoplasmic reticulum vacuolization and cardiac functional defects
Source: Nat Commun. 2020 Feb 19;11:965. doi: 10.1038/s41467-019-14143-9 (PMC7031342; doi:10.1038/s41467-019-14143-9)
Supplement: Supplementary file 14 — Description of Additional Supplementary Files [file 41467_2019_14143_MOESM14_ESM.pdf]

Title: Supplementary Movie 1.
Description: Control embryo at 96hpf (side view). Regular heartbeat of 1:1 (atrium:ventricle) contractions can be observed with normal blood flow and chamber orientation.

Title: Supplementary Movie 2.
Description: Control embryo at 96hpf (ventral view). Regular heartbeat of 1:1 (atrium:ventricle) contractions can be observed with normal blood flow and chamber orientation.

Title: Supplementary Movie 3.
Description: REEP5 MO embryo at 96hpf. Atrium:ventricle contraction rhythm has shifted towards a 3:1 ratio with minimal blood flow passing between chambers.

Title: Supplementary Movie 4.
Description: REEP5 1X gRNA injected CRISPR mutant at 96hpf. Regular heartbeat can be observed, and a synchronous atrio-ventricular contraction rhythm is maintained.

Title: Supplementary Movie 5.
Description: REEP5 2X gRNA injected CRISPR mutant at 96hpf. A marked reduction in heartbeat and the linear orientation of the heart from a lack of cardiac looping can be observed compared to controls (See Supplementary Movie 1). No ventricular contraction was observed.

Title: Supplementary Movie 6.
Description: REEP5 3X gRNA injected CRISPR mutant at 96hpf. Slower heartbeat compared to controls and asynchronous atrio-ventricular contraction rhythm can be observed with minimal ventricular contractions.

Title: Supplementary Movie 7.
Description: REEP5 1X gRNA injected CRISPR mutant shows reduced systemic trunk circulation at 96hpf. Left: REEP5 1X gRNA injected embryo, Right: control embryo.

Title: Supplementary Movie 8.
Description: REEP5 2X gRNA injected CRISPR mutant shows completely arrested systemic trunk circulation at 96hpf. Left: control embryo, Right: REEP5 2X gRNA injected embryo.

Title: Supplementary Movie 9.
Description: *reep5* CRISPR/Cas9 homozygous mutant appears phenotypically normal with regular heartbeat of 1:1 (atrium:ventricle) contractions.

Title: Supplementary Movie 10.
Description: *reep5* CRISPR/Cas9 homozygous mutant injected with REEP5 MO (1ng)

shows normal chamber orientation with regular heartbeat of 1:1 (atrium:ventricle) contractions.
